# How Do Scientists Perceive the Relationship Between Ethics and Science? A Pilot Study of Scientists’ Appeals to Values

**DOI:** 10.1007/s11948-023-00429-1

**Published:** 2023-04-25

**Authors:** Caleb L. Linville, Aidan C. Cairns, Tyler Garcia, Bill Bridges, Jonathan Herington, James T. Laverty, Scott Tanona

**Affiliations:** 1 Department of Philosophy, Kansas State University, 1116 Mid Campus Dr North 201 Dickens Hall Manhattan, 66506-0803 KS Manhattan, United States; 2 Physics Education Research, Department of Physics, Kansas State University, Manhattan, United States; 3 Department of Philosophy, University of Rochester, Rochester, United States

**Keywords:** Responsible Conduct of Research, RCR education, Research ethics, Values in science, Physics education research

## Abstract

Efforts to promote responsible conduct of research (RCR) should take into consideration how scientists already conceptualize the relationship between ethics and science. In this study, we investigated how scientists relate ethics and science by analyzing the values expressed in interviews with fifteen science faculty members at a large midwestern university. We identified the values the scientists appealed to when discussing research ethics, how explicitly they related their values to ethics, and the relationships between the values they appealed to. We found that the scientists in our study appealed to epistemic and ethical values with about the same frequency, and much more often than any other type of value. We also found that they explicitly associated epistemic values with ethical values. Participants were more likely to describe epistemic and ethical values as supporting each other, rather than trading off with each other. This suggests that many scientists already have a sophisticated understanding of the relationship between ethics and science, which may be an important resource for RCR training interventions.

## Introduction

One challenge to promoting ethical behavior in science is that scientists sometimes view ethics as being external to science (Hempel, 1965; Lacey, 1999; Betz, 2013). Douglas (2000) notes the influence of the long tradition of the “value-free ideal” that holds that value-laden decision-making about science is limited to the choice of projects and application of products in society. However, there is a growing set of analyses of science that suggest value questions cannot be ignored in science (Douglas, 2009; Brown, 2013, Biddle, 2017). These analyses suggest that ethical science requires attention to the consequences of decision-making at a variety of stages within science, and that what it means for a research project to be “good science” is not merely a matter of epistemic norms. On these views, ethics is *integral* to science, since good scientific methodology requires attention to both epistemic and non-epistemic values. Research fraud, for example, not only undermines the epistemic goals of science, but wastes future researchers’ time and funding, imposes opportunity costs, and risks harm to society through actions that might be based on falsified research. Even an apparently non-problematic methodological decision such as a choice of statistical method can carry ethical consequences, for example by influencing the ratio of false positives to false negatives.

Given these arguments, we hypothesize that knowledge of how scientists view the relevance of ethics to their work, and especially to their epistemic goals as researchers, is critical to efforts to promote ethical behavior in science. For example, do they view ethics primarily as external standards separate from methodological criteria for achieving epistemic goals such as accuracy? Or do they rather think of ethics as a way of working towards epistemic goals? While there is a body of research that suggests research ethics training is largely ineffective (Antes et al., 2010, Kalichman, 2014, Mumford et al., 2015), there is little work that investigates how we might improve ethical behavior with knowlege of how scientists use and move between science, ethics, and epistemic values.

This paper investigates how scientists think about the role of ethical values in science, and how they relate ethical values to other values, both epistemic and non-epistemic, by identifying the values scientists invoke in answers to questions about ethics in their own work and about research ethics vignettes.^1^ To that end, we investigate how scientists relate ethics and science in three ways: (1) we look into the types of values that scientists appeal to when faced with ethical questions; (2) we analyze how explicitly scientists relate epistemic values with ethical values; and (3) we explore the relationships that scientists express between different values.

## Theoretical Framework and Background

### Approach to Research and Learning

Modern theories of learning suggest that people actively make their own knowledge, building from what they have previously learned (Steffe & Gale, 1995). From this perspective, we argue that teaching scientists to engage in research more responsibly will be more effective if they are built around scientists’ existing ideas and practices. Such an approach assumes that individuals’ conceptions are made up of small pieces of reasoning (referred to as “resources”). Resources are neither right nor wrong by themselves, but may be properly or improperly applied within a given context (Hammer et al., 2005). Interventions built around these ideas focus on identifying individuals’ productive reasoning and providing them opportunities to apply and refine the application of that reasoning. This lies in contrast to many existing training programs for RCR, which focus on delivering information about ethical standards. In this paper, we work to identify resources that scientists use when reasoning about ethical concerns: namely, the values they invoke and the relationships between those values.

### Values in Science

The resources of interest for this study are the values of scientists that, together with their beliefs about the world and evidence they uncover, drive their decision-making. Hausman (2011) describes values in terms of preferences, wherein if a person prefers one thing to another, they value that thing. It follows from this that values are relational; things are being valued, and someone is doing the valuing. This further implies that there can be hierarchies of values, that some values might be foundational, and that a goal is a type of intentional value.^2^ Moreover, values can have different relationships to one another. Values can conflict; for example if a person would prefer to realize two things, but it is only possible to realize one. Values can be supportive as well; for example, if the achievement of one value serves as an instrument for the pursuit of some other.

Scientific values are the aims scientists try to achieve qua scientist and the things they prioritize in practice. Science is not a monolith, and these vary with discipline, institution, and even individual, down to the motives, incentives, and goals that drive scientists’ decision making. These values may include epistemic goals, personal aims, or ethical principles. Scientists might value truth as a general goal of science, and thus value the uncovering of particular facts as a specific goal for their research. They might have particular career interests. They might be motivated to do their work to help society.

### Prior Work on Scientists’ Beliefs About Values in Science

Which values may *appropriately* drive scientific inquiry, and how scientists should attend to non-epistemic values in the pursuit of science, has been a matter of debate. The so-called value-free ideal indicated that science itself should remain value-free, even if the reasons for doing that science may be value-driven. On this view, while scientific practice might be *constrained* by ethical rules and principles, and some choices of projects or applications might be value-laden, science itself pursues purely epistemic goals such as attaining knowledge, understanding, and truth. Responsible conduct of research might require behaviors such as good record-keeping for the purpose of furthering science, but responsible conduct here is an instrument for epistemic goals rather than for independent ethical reasons.

Douglas and others (e.g., Douglas, 2009; Brown, 2013; Biddle, 2017) suggest that this view is mistaken, that the value-laden implications of decisions are unavoidable, and that scientists should more actively consider non-epistemic values. For example, Douglas’s (2000) examination of dioxin studies on rats demonstrates how decisions throughout research can affect the balance of the “inductive risk” of wrongly accepting or rejecting a hypothesis, with correspondingly different potential effects on human health. Douglas argues that scientists’ decisions have unavoidable ethical dimensions and are value-laden whether scientists attend to those values or not. She further argues that the appropriate response to the recognition of the impact of those choices is to explicitly attend to those potential consequences in at least some stages of scientific decision-making. The suggestion that value-ladenness is unavoidable picks up on a range of literature suggesting the inevitable entanglement of science and values (e.g., Rudner 1953; Graham, 1979; Kuhn, 1996; Myrdal, 1970).

More recent values-in-science literature identifies different approaches to the involvement of non-epistemic values. While Douglas (2000, 2009) suggests that the reality of inductive risk undermines the distinction between epistemic and non-epistemic values, she argues for only an indirect role of values, where the ramifications of wrongly accepting or rejecting a hypothesis should be considered in the choice of methods, but valuations of potential consequences should not be used directly to determine conclusions (e.g., data should not be rejected because they do not favor a hypothesis). Steel (2010) retains the distinction but argues that non-epistemic values should have a role in scientific inquiry because of inductive risk; he claims that social costs associated with acceptance or rejection of hypotheses should determine evidential thresholds. Other theorists hold that non-epistemic values should be restricted from science. For example, Lacey (1999) argues normatively that science should be value neutral, and that theories should be evaluated solely on epistemic merit, and Resnik and Elliot (2019) suggest that rejecting the value-free ideal risks undermining the integrity of scientific research.

While there has been considerable normative and philosophical work on values in science, empirical studies of what scientists think of the role of values in science are more limited. While there has been some discussion (O’Rourke & Crowley 2013; Robinson et al., 2019; Beebe and Delsén, 2020; Schindler, 2022) about the relative importance scientists place on different, mostly epistemic, values, few focus on both epistemic and non-epistemic factors.^3^ However, there is evidence that some scientists recognize non-epistemic values as having a place in scientific inquiry. For example, Steel et al. (2018) found that the value-free ideal is not an unequivocally dominant viewpoint. They found a tendency for scientists in their survey to hold that science could be objective and guided by societal values simultaneously. They also found that participants that identified as female and participants in non-natural sciences were more likely to depart from the value-free ideal. We extend this research by identifying the types of values that scientists invoke specifically in the case of ethical questions. If scientists generally hold to the value-free ideal, then we should mostly see appeals to epistemic values. However, if they do not hold that view, then we should expect to see a mix of epistemic, ethical, and other values.

Some studies suggest that scientists view science as being constrained by certain non-epistemic values. Kempner et al. (2005) states that scientific inquiry might be restricted because certain questions can only be addressed using unethical means. Additionally, non-epistemic concerns, such as social pressure and criticism, might restrict science in concert with ethics. Wolpe (2006) claims that scientists might avoid thinking about ethics because they view ethics as arbitrary restrictions. We want to see if scientists articulate epistemic and ethical values as being in positive or negative relationships with each other. If scientists consistently describe ethical and epistemic values as trading off with each other, that could imply that scientists see ethics as a restraint on science. If they see them as supportive, we want to know how and in what ways: e.g., do they view research ethics primarily as something needed to help achieve epistemic goals as a community?; alternatively, does potential benefit to society motivate epistemic values of science?

Pennock and O’Rourke (2017) suggest a value approach to integrating ethics into science using the concept of scientific virtues, character traits that are conducive for achieving the goals of science. They claim that scientific virtues can be implemented through theory-centered, exemplar-centered, or concept-centered methods. We look at a broad range of values, including scientific and epistemic virtues together with explicitly ethical and social values, as well as how scientists relate them. A better understanding of how scientists relate values can help us identify appropriate methods for integrating ethics in science.

## Methods

### Research Questions

We identified three research questions.

1. What *types* of values do scientists appeal to when reasoning about ethics?
2. What types of values do scientists *explicitly associate* with “ethics”?
3. How do scientists *relate* ethical and epistemic values?

### Definitions of Categories

We defined eight different types of value, utilizing a range of existing definitions in the literature. Following Steel (2010), we separate epistemic and non-epistemic values. For our purposes in this study, we construe the epistemic category broadly to include not only appeals to truth and other empirical concerns, but also to values like simplicity and explanatory power. Non-epistemic values include all other values that may influence scientific decision making.

We differentiate non-epistemic values into several categories (see Table 1). Some of the categories (Ethical, Communitarian, RCR/Legal, and Self-interest) are based on Rest et al.’s (2000) neo-Kohlbergian model of ethical reasoning, which differentiates between reasoning based upon personal interest, maintaining norms, and postconventional moral values. While we reject a strict ordering of values, we want to differentiate between broadly ethical values such as explicitly utilitarian or deontological values and other types of other-directed values such as reciprocal or rule-following reasoning. Following the general line that postconventional thinking is more properly identified as “ethical”, we differentiated communitarian values from ethical values in order to distinguish ethical motivation from the desire for social approval. We also included a category to capture cases where researchers valued maintaining institutional and legal standards, including “RCR” rules of conduct set by professional or granting agencies. The category of practical values was introduced after we noticed that interviewees would appeal to values that were related to furthering other goals, but were not clear about the nature of the further goals; all other categories had been defined prior to coding. Subcategories of values were developed in the first pass of categorizing, to allow more fine-grained analysis. A more detailed list of categories and their definitions can be found in the Appendix.

**Table 1:** Definitions of Categories of Value Appeals

Category	Definition
Ethical	An appeal to moral qualities, such as benefit to society or respecting others’ autonomy. We distinguished ethical values from those specifically about following law, tit-for-tat reasoning, or merely getting along with others. Subcategories: Ethical, Rights, Fairness, Equality, Social Good, Virtue, Interpersonal Care
Epistemic	An appeal to knowledge, truth, empirical adequacy, or understanding, or to methods for achieving these. Subcategories: Epistemic, Alethic, Explanatory, Understanding, Methodological, Aesthetic, Predictive, Empirical, Applications, Objectivity
RCR/Legal	An appeal to following laws or rules set by a governing entity, or by guidelines set forth by an RCR body. Subcategories: RCR, Legal
Communitarian	An appeal to peer/social approval, or general civility or comity, e.g., avoiding conflict in small groups. This is not an ethical category, since it is ambiguous whether the rationale for maintaining social order is based in self-interest or the interest of others. Subcategories: Peer approval, social approval
Economic	An appeal to resources such as time or money, or management of limits thereof.
Self-interest	An appeal to some benefit to the interviewee.
Practical	An appeal to something that is not an epistemic concern, but is a necessary prerequisite for doing science.

The seven categories of values and their definitions. Definitions of subcategories are in the Appendix.

### Context

Our data comes from interviews with scientists that took part in a year-long fellowship program oriented around improving RCR by using explicit discussions of the values of science. This fellowship occurred at a large midwestern university, and had fifteen participants and one facilitator. There were six participants from the discipline of biology, three from chemistry, three from physics, two from biochemistry, and one from geology. Three of them were female, and twelve were male. All participants held tenure-track positions and eleven of the participants already had tenure. Participants were recruited using a snowball methodology involving email, word of mouth, recommendation, and explicit invitations by the fellowship organizers. Recruitment intentionally tried to promote diversity in terms of gender, race, and academic status.

The fellowship consisted of meetings throughout the academic year that focused on goals and values of science, such as the relative priority of truth, predictive accuracy, and social benefit, or the consequences of choices regarding statistics. Participants were interviewed about ethics both before and after the fellowship. Interviewees were told that the interview was not a test to judge whether they were behaving ethically, but rather to learn how they reason with ethical and epistemic values. This study focuses on the pre-fellowship interviews.

### Data Collection

Data were collected from fifteen interviews with science faculty focused on the relationship between ethics and science. The interviews took place before the fellowship sessions. The interviews were conducted in private by one of the authors, who is a male philosophy professor. Two of the participants had had prior professional interactions with the interviewer through an education training project; two had had previous personal interactions with the interviewer; and two had prior knowledge of the interviewer. The interviews took place either in the campus office of each participant or (in one case) in the interviewer’s office, and were video and audio recorded. Field notes were taken during the interviews, but not used in this study. All of the interviewees responded to the same interview questions, divided into two main sections: the first featured questions about their general experience with ethics in their own careers, and the second asked questions about fictional research ethics vignettes (see Table 2). We included each set of questions to see how scientists reason about ethics in both their direct experience and hypothetical dilemmas. Additionally, the vignettes were designed to elicit thinking about tradeoffs. Transcripts were not returned to participants for correction or revision after the interviews.

There were four questions about the interviewee’s general experience with ethics, and three fictional vignettes. Two of the vignettes were adapted from the Ethical Decision Making Measure (Mumford et al., 2006) and had subparts which focused on a different RCR topic. In total, there were six topics. The four questions and the topics in each of the three vignettes are listed in the tables below. In the portion of the interview focused on general experience, follow-up questions were asked based on the responses to the original questions. In the section with the vignettes, a fictional vignette would be presented and then the interviewee was asked what they would do in that situation.

**Table 2:** Summary of the Interview Questions

Omitted first question	What is the focus of your research?	
General Experience	What does ethics mean to you?	
	What kinds of ethical issues do you run into in your research?	
	What about publishing? Do you run into any ethical issues in that area?	
	What about working with other people? Any ethical concerns there?	
Vignette Topics	1st Vignette	Informed Consent
		Suspicious Data
		Authorship Issues
	2nd Vignette	Conflicts of Interest
		Inappropriate Research Inspiration
	3rd Vignette	Diversity

Responses to the first question were not analyzed in this study.

### Data Coding

All fifteen interviews were transcribed using an automated transcriber (otter.ai). The transcripts were updated for accuracy by the coders as needed during the coding process. Coding was performed by viewing the videos alongside the transcripts. We first coded all fifteen interviews based only on the general experience questions, and then coded the vignettes questions. The answers to one interview question were omitted, since it asked about the interviewee’s research in general. That question did not address our larger inquiry, since it was only about research, and not about ethical issues.

While viewing the interviews, coders looked for statements that implied the interviewee valued something or had a goal of achieving something. Since a value is something that guides or motivates actions, coders looked for statements where the interviewee stated that they did, would, tried to, wanted to do something, or simply desired or preferred something. We operationalized this process by fitting the value into the sentence “The interviewee has a goal of doing X,” or “The interviewee cares about X.” By operationalizing in this way, we ensured that we identified objects of valuing rather than general statements about the world, and avoided coding descriptive statements made by the interviewees. The search for value statements relied solely on the videos of the interviews, which would be watched twice. When we identified a value that fit into one of those sentences, we identified the surrounding quote in the transcript, fixed the transcribed quotation as necessary, and then copied that quotation into a spreadsheet to document it. Often, the quotations were parts of a longer monologue by the interviewee; when this was the case, the entire the entire discourse was not included in the spreadsheet. We included enough of what the interviewee said to give appropriate context for the value or goal being appealed to, and we ended the quote when either the interviewer started talking, or when the interviewee shifted to a different subject.

The specific values we identified from the quotes were documented alongside them in the spreadsheet, along with time stamps. The values were documented to be as close to the original wording of the interviewee as possible. We avoided using synonyms, and we also avoided paraphrasing, over-summarizing, or inferring implicit values. When the interviewee expressed a value not actually held by her or him (for example, one interviewee spoke about one of his students valuing precision), we did not include those values in the analysis because their status as motivation for the scientist was unclear. A quote could have multiple values. Examples of this coding can be found in Table 3.

When an interviewee related one value to another, the two values would be documented separately, with a relationship indicating how the interviewee indicated they were connected. We used three different relationships: supporting, tradeoff, and prioritization. Supportive relationships were documented when the realization of one value was conducive to the realization of another value. Tradeoffs were documented when the interviewee indicated they held two values that could not be realized simultaneously. Prioritization relationships were documented in the case of a tradeoff where the interviewee indicated one value should be prioritized over the other. If the interviewee did not express a relationship between multiple values, we simply listed the values. Examples of relationship coding can be found in Table 4.

We also noted when the interviewee explicitly associated values with ethics. When an interviewee was directly responding to the question “What does ethics mean to you?” or when they explicitly stated something was an ethical issue, we noted it. We wanted to capture these views in order to better understand how faculty thought about ethics.

An example of a value that the interviewee explicitly called ethical is “I think what I mean by that is, so, ethics is so fundamentally it’s like, do no harm or you know, work towards a common good.” In this case, we can infer the values of avoiding harm and working towards the common good, which are both ethical values. Another interviewee responded to the question “What does ethics mean to you?” with “Not falsifying data. Making sure you’re reporting accurate findings. Making sure your work is rigorous and reproducible. That sort of thing.” This interviewee answered with epistemic values.

### Data Analysis

After documenting the quotes, values, and times, we placed the values into the theoretical categories defined in the Methods Section. These categories are Epistemic, Ethical, Communitarian, Self-Interest, RCR/Legal, Practical, and Economic. Each category had several subcategories (for example, the category Ethical was divided into Rights, Fairness, Equality, Social Good, Virtue, and Care). All of the categories and their subcategories can be found in the Appendix. When placing a value into a category, we placed it into a subcategory first, which was tied to the larger category. To determine the category both the value itself and the quote that it was extracted from must be considered. We categorized the values based on how the interviewee explained the motivation for holding that value, which could be found in the quote from which the value was extracted.

**Table 3:** Examples of coding and categorization of quotes from the interview data

Quote	Value	Subcategory	Category
“I think it is important to understand that some things might change over time, and are context dependent, while others may or may not. **So I think fundamentally, do no harm.** Ideally, starting with your own species.”	Avoiding harms	Ethical	Ethical
“I would just say for me, it’s kind of like the **fear of like, like kind of putting something out there that’s false.”**	Avoiding making false claims	Empirical	Epistemic
“He should **state a conflict of interest with the program officer and recuse himself from that review committee**… Any review that he gives of that proposal will clearly be biased by his own results and his own hypotheses of the work.”	Avoiding conflicts of interest	RCR	RCR/Legal
“You know, if you’re collaborating with somebody, chances are at least in that scenario that I see, probably the PIs, or the, you know, the senior personnel probably view things from exactly the same point of view because they’re working together. Yeah, and work with somebody who seems antagonistic to your perspective on what’s going on? Yeah, sounds like a good case for having a lab meeting or something like that to kind of, you know, **make sure everybody’s sort of on the same page.”**	Having everyone on the same page	Social Approval	Communitarian
“At some point we’re going to have to like, submit this paper and see what the reviewers say… and find out what somebody else thinks about the experiments, and then we’ll, you know, decide if we’re going to do more or not because, you know, this also costs money, you know, to do it, and **we were not time unlimited, we’re also not funding unlimited.”**	Time and money as a resource	Economic	Economic
[How did you feel about that?] “Well, I still feel like I wish the postdoc had talked to me. **Of course, there’s a little bit of your own reputation on the line**. At the same time, it’s an article that probably nobody is going to really read.”	Maintaining one’s own reputation	Self-Interest	Self-Interest
“For example, tomorrow we’re going to be, a student and I, downstairs, cleaning a lab space. Like, basically getting it ready. We have to do this periodically. One of the things we have to do with these rocks in order to understand their chemistry is to crush them up, pulverize them, or powder them. **You want to start with a clean facility, or at least as clean as possible**…So it’s stuff like that, which is not at all, you know, doing the science, but you’re preparing to do the science, versus you know, doing maybe data interpretation for the sample.”	Keeping labs clean	Practical	Practical

The specific part of the quote that the value is pulled from is in bold.

For example, one interviewee said:

Oftentimes we say that science is really about trying to find the truth about how things work. And so, if you’re not doing things in the right way or in the proper way, you can definitely get the wrong results, or actually cause you to go down a path that’s not right and proper. If you’re simply about getting the results instead of actually getting the truth out of the results, then that can lead to unethical behavior.

From this quote, we pulled four values and two relationships. First, the interviewee implies that consequences can arise from not doing things in the right or proper way; we coded the value of “Doing things in the right way,” and categorized that as Ethical. Second, he says that doing things in that way can cause you to get the wrong results. We coded this as “Getting the right results,” which was categorized as Epistemic. Those two values are related to each other, since he implies that doing things in the right way supports getting the right results.

The interviewee also claims that it is a mistake to care more about simply getting results than about getting truth from the results. We coded the value of “Getting the truth out of the results,” and categorized that as Epistemic in the subcategory of Alethic. We also coded the value of “Simply getting the results,” which was categorized as Epistemic. These two values were connected by the relationship of “Getting the truth out of the results” being prioritized over “Simply getting the results.”

**Table 4:** Examples of Relationships

Relationship	Quote	Values	Categories	Subcategories
Tradeoff	Even the ethical decision of do I want to do fundamental research, Or **do I want the immediate impact of your own contribution?** Right? So how can you make this decision? You know, there are a million people dying of mosquito borne diseases roughly every year, **how can I sit here and do basic fundamental research?**	Doing fundamental research trades off with immediate medical impacts	Epistemic trades off with Ethical	Understanding trades off with Social Good
Supportive	In some sense, an important part of science is that we check each other’s work constantly. **If I don’t supply sources, it is harder to check the whole line of my research.** If I deliberately leave someone off, in terms of credit, I might be doing that person some damage, but I might be doing far more damage by not providing a track that people could work back along. They could only check my work, they couldn’t check what my work was based on.	Giving proper credit supports checking science	RCR supports Epistemic	RCR supports Epistemic
Prioritization	I basically said, look, the fundamental, the underlying, or overriding issue here is academic freedom. This person’s exercise of **academic freedom is more important to our field than whether someone else’s feelings are being hurt.**	Maintaining one’s right to academic freedom is prioritized over not hurting others’ feelings	Ethics is prioritized over Communitarian	Rights are prioritized over Social Approval

The specific part of the quote that the values and relationship are pulled from is in bold.

Another interviewee said:

Ethics, um, that’s a good question. I mean, as a working scientist, I think it has a lot to do with reproducibility of results. Like if we’ve said we’ve done this experiment, and we get this result, I should have some sort of confidence that we’ve described the experiment well enough, and that we sort of understand the sources of error and the like well enough, that if someone else sets out to reproduce our work they should be able to.

From this, we noted first that the interviewee is explicitly referring to ethics in this quote. He says that ethics has a lot to do with reproducibility, so we coded the value as “Reproducibility,” which was categorized as Epistemic in the subcategory of Methodological, with an explicit association with Ethics.

After categorizing the data, we ran an inter-rater reliability check for the categorization of the values. The checks on categories were only done on values that had been agreed on by all coders. Inter-rater reliability checks on categories were done with three coders. For these checks, one coder would send a series of quotes that he or she had analyzed. The two other coders would analyze the quotes and record their categorization of the interviewee’s values. The original coder would compare their work to the other coders’ work, and then all the coders would meet and discuss which categorizations to accept when there was a disagreement. The categories were predefined, and all coders used the same list. Inter-rater reliability checks were done on 45 values; the Fleiss’ Kappa value was 0.89, which signifies “almost perfect agreement” (Landis & Koch, 1977).

After categorizing the values in the spreadsheet, we calculated the frequency and relative percentage of each category, as well as the subcategories. We calculated these for the fifteen interviews. Additionally, we calculated the frequency of different relationships, as well as the two value categories that they tied together. We did these calculations twice; once with all of the values, and once with only the values with explicit association with ethics. Participants did not provide feedback on findings.

## Results

**Fig. 1:**
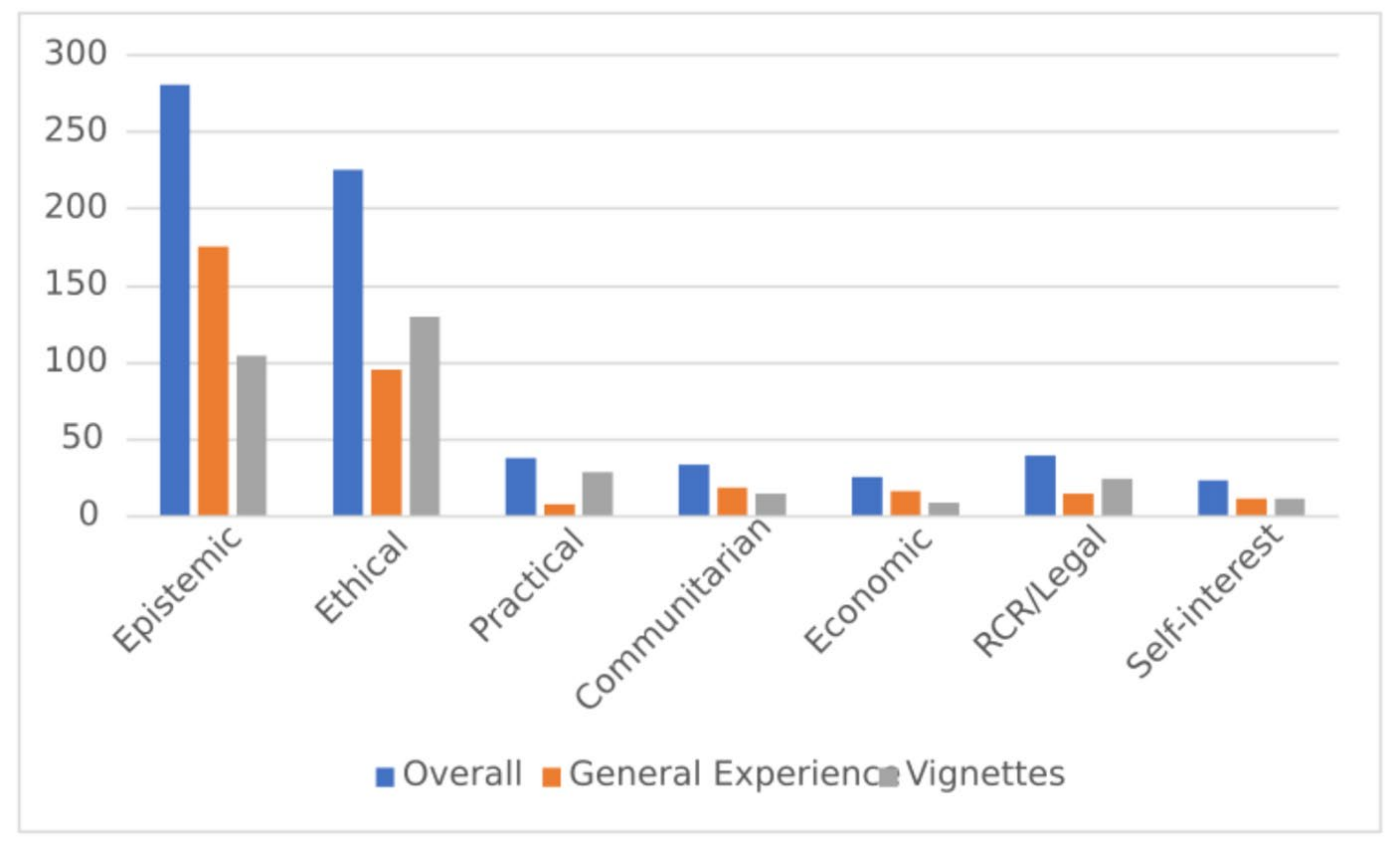
Number of Appeals to Values by Category

As seen in Fig. 1, the two largest value categories are Epistemic and Ethical. All of the other categories accounted for markedly fewer value appeals than the Epistemic and Ethical categories. Overall, epistemic values were appealed to more often than ethical values; however, in the vignettes, slightly more ethical values are appealed to than epistemic values.

Epistemic values appealed to includes such goals as reproducibility and confidence about data being reported, such as in this example:

*“Until we know exactly what’s happening, we just call that the end of the line. So if we aren’t 100% certain on what we believe, the results, and the data we have is repeatable and conclusive, we won’t publish at all.”*

Ethical values expressed often appealed to betterment of society or avoidance of harm, such as in this example:

The right thing means, not just for me to profit, but for humanity to be better off, and for sure, for humanity not to suffer from what I do.

The quantities of each category are shown in Table 5.

**Table 5:** Number of Appeals to Values by Category

Category	Overall	General Experience	Vignettes
Epistemic	281	176	105
Ethical	226	96	130
Practical	38	8	29
Communitarian	34	19	15
Economic	26	17	9
RCR/Legal	40	15	25
Self-interest	24	12	12

**Fig. 2:**
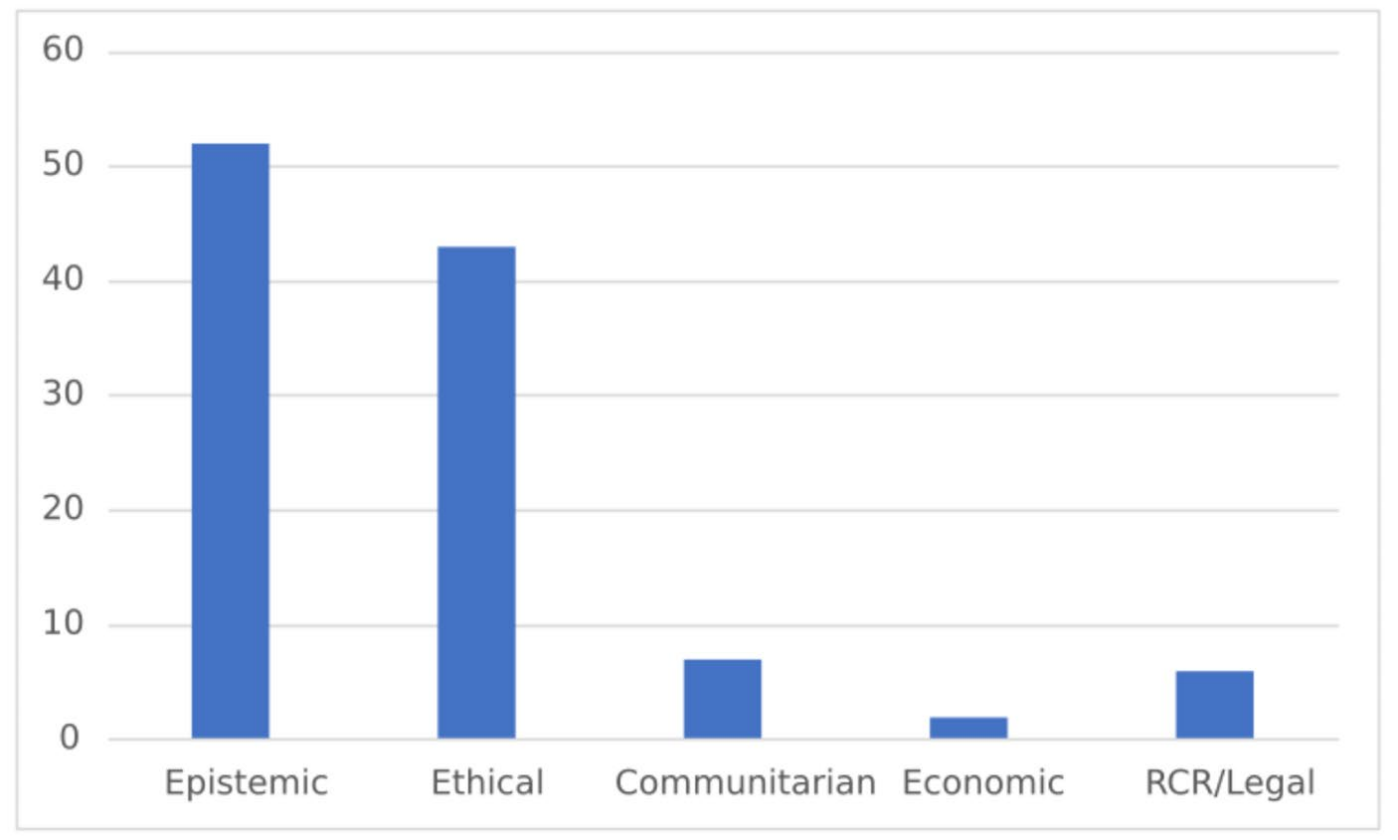
Number of Appeals to Values Explicitly Associated with Ethics by Category

Epistemic and Ethical are also the largest categories of values that interviewees explicitly associated with ethics (see Fig. 2). Notably, epistemic values accounted for the largest category when only considering these values. The interviewees explicitly denoted practical values and self-interested values as ethical zero times. These numbers are reflected in Table 6.

An example of epistemic values explicitly associated with ethics is found in the following quotation:

It would be unethical to disseminate information that you’re not confident is reliable… I think it’s our responsibility to report data that we have seen consistently, and, not necessarily, this goes along with not falsifying, you know, maybe massaging data, to complete your story, the hypothesis that you love.

The values expressed by the interviewee are epistemic: reliability, repeatability, and truth of the data. However, the quote begins framing these as ethical concerns, indicating that the epistemic goals are of ethical value.

**Table 6:** Number of Appeals to Values Explicitly Associated with Ethics by Category

Category	Number of Appeals
Epistemic	52
Ethical	43
Practical	0
Communitarian	7
Economic	2
RCR/Legal	6
Self-Interest	0

**Fig. 3:**
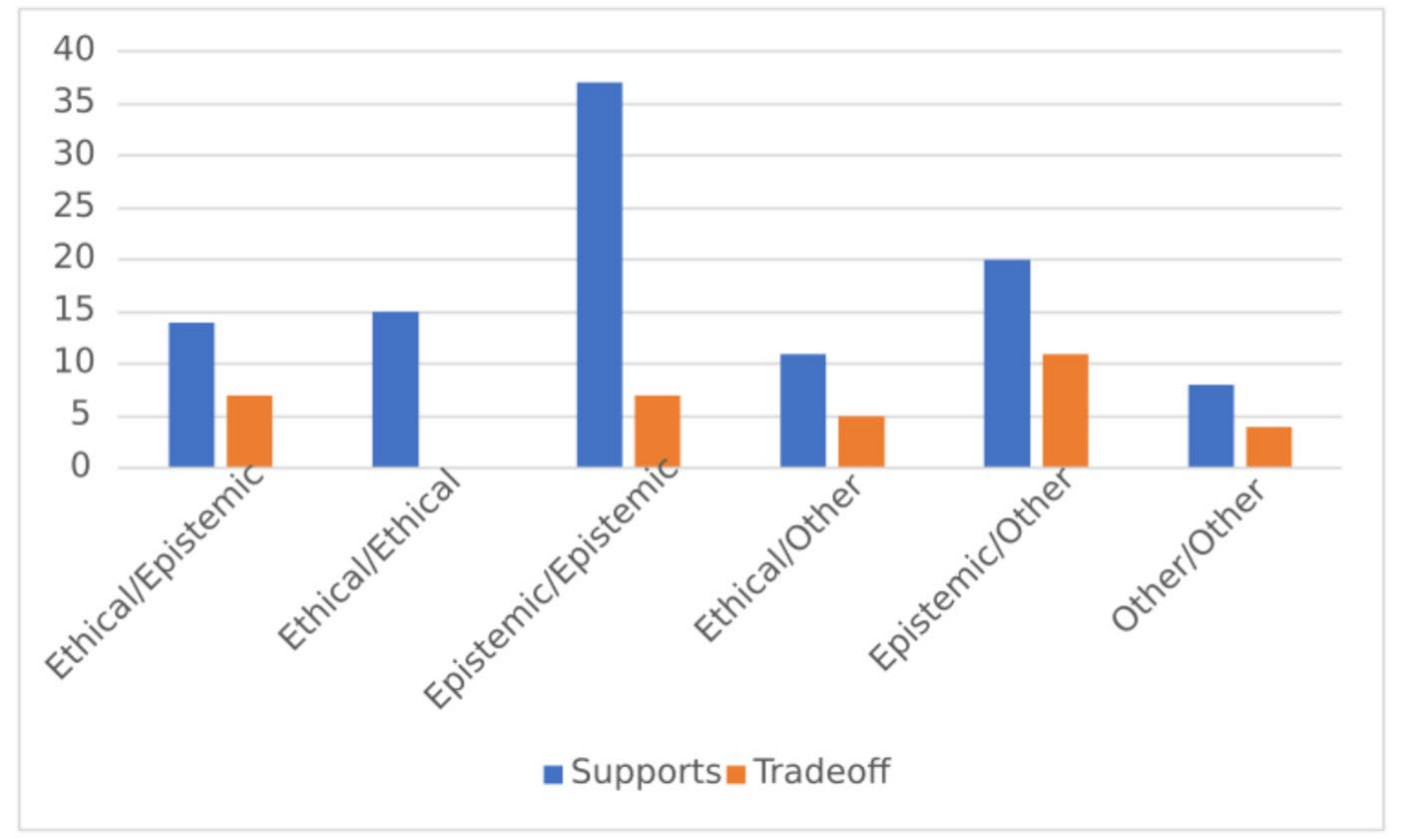
Relationship between Value Categories

Scientists in the study expressed more supportive relationships between epistemic and ethical values than negative relationships (see Fig. 3): 10.1% of values were connected by tradeoffs, and 31.4% were connected by supportive relationships, for a total of 41.5% connected by some relationship. The most frequent supportive pair were epistemic values with other epistemic values. Ethical values were expressed as supporting epistemic values at a similar rate as ethical values supporting other ethical values. Fewer tradeoffs were expressed than supportive relationships; the interviewees mentioned fewer examples of ethical values trading off with epistemic values than those types of values supporting each other, and zero instances of ethical values trading off with other ethical values were mentioned. Counts and percentages of the relationships between value categories can be found in Table 7.

Tradeoffs between epistemic and ethical values were expressed seven times. For example:

This is a truth-seeking exercise. And getting the truth out is more important than maintaining someone’s ego.

The interviewee here was stating that the epistemic value of truth-seeking should be prioritized over the ethical value of caring for others’ emotional state, suggesting that in some circumstances the two values might conflict.

Another example:

I think there’s all sorts of interactions about how you interact socially and professionally with the postdocs and grad students, you know how do you manage, you want to be pushing their research productivity, but you don’t want to be making them miserable either.

The interviewee here expresses an ethical value of avoiding harm to postdocs/graduate students, and an epistemic value of maximizing research productivity. In this example, the interviewee does not indicate that one value should always be prioritized over the other, but just indicates that they could come into conflict.

On other occasions, interviewees indicated that ethical values supported epistemic aims. For example, one interviewee said the following:

There is an assumption anytime you submit a grant that it will be reviewed without bias, and that system only works when there is voluntary reporting of bias. So it’s kind of a greater-good scenario, where if you want the granting system to work you have to report times when it would be advantageous for you to act.

Here the voluntary reporting of bias is an ethical, “greater-good” value that supports the epistemic value of having a bias-free grant reviewing system.

**Table 7:** Relationships Between Value Categories

Value-Value Relationship	Relationship Type	Number of individuals expressing	Number of times expressed	Percentage of Value-Value Relationships	Percentage of Total Relationships
Epistemic-Epistemic	Supportive	13	37	84%	27%
	Tradeoff	5	7	16%	5%
Epistemic-Ethical	Epistemic supports Ethical	5	7	33%	5%
	Ethical supports Epistemic	6	7	33%	5%
	Supportive (total)	10	14	66%	10%
	Tradeoff	6	7	33%	5%
Ethical-Ethical	Supportive	5	15	100%	11%
	Tradeoff	0	0	0%	0%
Epistemic-Other	Epistemic supports Other	1	1	3%	0.7%
	Other supports Epistemic	9	19	61%	13%
	Supportive (total)	10	20	64%	14%
	Tradeoff	6	11	36%	8%
Ethical-Other	Ethical supports Other	2	3	19%	2%
	Other supports Ethical	6	8	50%	6%
	Supportive (total)	7	11	69%	8%
	Tradeoff	5	5	31%	4%
Other-Other	Supportive	5	8	67%	6%
	Tradeoff	3	4	33%	3%

‘Value-value relationship’ refers to possible combinations of value categories interacting with each other; for example, ‘Epistemic-Ethical’ refers to epistemic and ethical values interacting with each other.

## Discussion

These results allow us to address our three research questions: what types of values do scientists appeal to when talking about ethics; which of those values do they explicitly associate with ethics; and how do they relate epistemic and ethical values?

The scientists in our study reasoned about ethical problems using *both* ethical and epistemic values. We observe that the appeals to epistemic values and appeals to ethical values occur at roughly the same frequency, and that these two categories account for substantially more appeals than any other category. These overall patterns appear in both the questions about researchers’ general experience with ethics and the questions about the fictional vignettes. These patterns suggest a model of approaching research ethics not merely in terms of applied ethical principles, but also in terms of epistemic issues. We also found that scientists in this study rarely invoked legal or regulatory ramifications when considering research ethics. Grouped together, appeals to legal values and RCR rules only accounted for 4.4% of all value appeals. Together, these results cast doubt on the view that scientists view ethics as being external to scientific practice.

These were the values appealed to in reasoning about ethical problems. As to which values were explicitly associated with ethics, we found that the scientists in our study explicitly associated epistemic and ethical values with ethics more frequently than other types of values. This suggests that epistemic values are not just employed in ethical reasoning, but that ethical and epistemic values might be conceptually linked, and that their association might be available as a resource for research ethics training.

With regards to the question of how scientists relate epistemic and ethical values, our data do not support the view that scientists view ethics as a restraint on science, as suggested by Kempner et al. and Wolpe. Our sample expressed more examples of ethical and epistemic values supporting each other than trading off with each other. However, our study did not indicate whether this supportive relationship generally was directional, with ethics viewed more as an instrument for producing epistemically sound results or epistemic soundness viewed more as an ethical instrument for good.

Our study also did not uncover much explicit recognition of value tradeoffs in general. Understanding where and how values trade-off one another (for example, between autonomy and common good in many ethical dilemmas) is a critical aspect of good value-laden reasoning, but this type of reasoning did not appear in our study as a clear available resource for use in research ethics training.

### Implications

Although our study is only a preliminary investigation of scientist’s value-laden reasoning, if its results prove to be robust, they have a number of implications for efforts to promote research ethics. First, the relatively low rate of appeal to regulatory reasoning suggests that regulations and authority might not be a substantial part of researchers’ individual decision-making; if scientists were primarily motivated by impositions of authority, then, all things equal, they would be expected to refer to regulatory guidelines at a higher rate than other factors. If it is not, appeals to institutional ethics rules in education or practice will fail to engage with scientists’ common resources for ethical reasoning. Ethics should not be presented as a set of rules or guidelines external to science, since our evidence suggests that scientists do not perceive ethics as relating to scientific inquiry in that way. The more common association of ethical and epistemic values suggests an alternative resource for research ethics training.

Since research ethics promotion should be most effective when it allows scientists to employ resources they already have in their thinking about ethics and science, research ethics training should recognize both epistemic and ethical values. Failure to engage with epistemic values, for example by focusing only on general ethical principles or a set of standard RCR guidelines, misses the opportunity to elicit the epistemic dimension of ethical reasoning scientists already have. The association of ethical and epistemic values suggests a benefit to emphasizing the importance of good research practice for both epistemic and ethical reasons. Connecting ethical and epistemic reasoning can also help make explicit the value science has for society and the responsibilities of scientists have in virtue of potential for societal good or harm.

To make use of this resource, efforts to ensure responsible conduct of research can offer narratives of how research ethics relates to epistemic aims, such as emphasizing the epistemic value of good record-keeping, and the social value of finding the truth. For example, in a discussion of plagiarism, instead of focusing on its deceptiveness or unfairness to those that actually did the work, a program might invoke discussion about its effect on the ability of others to trace the progression of ideas, and how that might hinder other scientists. Such a discussion would invoke resources scientists already have, of thinking about research ethics in epistemic terms, and could make explicit the distinctive responsibilities of scientists. It also would allow entry into more complex, entangled value-laden discussions found in the values-in-science literature, such as how decisions about tradeoffs between false positives and false negatives might depend on consequences of each in specific contexts, rather than by choosing a standard level of significance.

These possibilities suggest a promising research avenue of exploring the effect of stimulating scientists’ reasoning about both epistemic and ethical values. More work is needed on how responsible conduct could be promoted by improving and building on recognition of value relationships.

### Limitations

There are some limitations on what can be inferred from our data. This study was done on a small sample and selection bias was possible. There were more men than women in the sample, which is notable in particular given the evidence of a correlation between gender identity and commitment to the value-free ideal (Steel et al., 2018). The *relative* importance scientists place on certain values cannot be properly inferred from the counts of the value categories, since the frequency of the categories is dependent on the structure of the individual interview, and there is some interpretation required to assign categories to statements. Similarly, no study of this kind can identify what the interviewees actually care about; we can only partially infer how they construct their reasoning according to the values they invoke in response to the general questions and hypothetical vignettes. As with any interviews on culturally-loaded topics, there is a possibility of social-desirability bias; interviewees might give answers that they think others want to hear. To reduce this effect, it was made clear to interviewees that the interview data would be anonymized and that the interviews were not tests of their ethicality. We also note that the coding is not an exhaustive list of the values that scientists appeal to: different scientists may appeal to a further set of values. Further research at a larger scale is therefore needed to establish the robustness of these results.

## Conclusion

After conducting interviews about ethics with fifteen science faculty members, we analyzed the interviews for expressions of values. The values were identified by the scientist’s expression of a goal or ideal that they would like to realize. Those values were categorized as ethical, epistemic, economic, communitarian, RCR/legal, self-interest, or practical, and the frequency of the categories was calculated. We found evidence that scientists think of ethical concerns in research in terms of a range of values, rather than only in terms of ethical values, with epistemic and ethical values as the two categories most appealed to. Additionally, we found that the scientists in our study explicitly associated both epistemic and ethical values with ethics. We found little evidence that suggests scientists think about ethical problems in research in terms of legal ramifications. We also found that scientists frequently think of epistemic values and ethical values in terms of supportive relationships. This suggests that research ethics programs should potentially focus on the justificatory role of both epistemic and ethical values in responsible conduct of research.

## Data Availability

De-identified, coded data is available upon request to the corresponding author.

## Appendix: Lists of Categories

### Types of Values

- Straight ethical: A value is appealed to because of what the interviewee thinks is the right thing to do, and it nearly always references some effect on another person.
  - Rights (autonomy, respect, rights): The appeal is based on treating people in a certain way because of some intrinsic feature.
  - Fairness/equality (fairness, equality, esp re distributions of goods): The appeal is based on treating each person in the same way.
  - Welfare/Social good (“good for people”, “improves lives”, “saves lives”): The appeal is based on doing something that will benefit society or a group of people, without any specific mention of the individuals that will be affected.
  - Virtue (e.g., honesty, generosity, trustworthiness, character): The appeal is based on fulfilling some character trait that the interviewee believes to be desirable.
  - Interpersonal care/pro-social (“need to listen to people in your lab”): The appeal is based on the interest of another person; the interviewee has a desire to maximize the welfare of a specific person.
- Standard RCR (credit for authorship, falsification, plagiarism, conflicts of interest, etc.): A value is appealed to because of regulations and guidelines set forth by RCR training.
- Legal/regulatory (“it’s important we follow IRB rules”, “we have to follow biosafety laws”): An appeal is based on specific threat of punishment or sanction by a governing entity.
- Communitarian: An appeal is based on the desire for peer/social approval. This would primarily involve actions to avoid conflict in small groups. It is not an ethical category, since it is ambiguous whether the rationale for maintaining social order is based in self-interest or the interest of others.
  - Social approval: An appeal is based on getting approval from a given community.
  - Peer approval: An appeal is based on getting approval from one’s peers.
- Scientific/epistemic: An appeal is based on advancing or improving science in some way.
  - Alethic (e.g. “the truth,” “know): An appeal is based on pursuing or clarifying knowledge or truth about something.
  - Explanatory/Understanding (e.g., “explain reality”, “understand our world”): An appeal is based on understanding some process.
  - Methodological (e.g. control, falsifiability, testability, reproducibility): An appeal is based on some specific part of the scientific method.
  - Aesthetic (e.g., “elegant theory”, “beautiful result”): An appeal is based on having a theory or explanation of some scientific phenomenon that is pleasing in some way.
  - Predictive (e.g., predicts, forecasts,): An appeal is based on conducting science in order to make predictions about the future.
  - Empirical (data-driven, open data, sharing information): An appeal is based on either the dissemination of data, or the proper use of data.
  - Technological/Applications: The appeal is based on using science to have some technological application, but no explicit other use or societal application.
  - Neutrality/Objectivity.
- Economic (i.e. “use resources wisely”, “grow the economy”): The appeal is based on using somewhat fixed resources.
- Self-interest (e.g., “get tenure”): An appeal is based on some benefit to the interviewee.
- Practical (clean labs, preparedness, … --things that are instrumental for science generally, but without specific other categories): An appeal is based on actions that are otherwise value-neutral, but must be done in order for science to be conducted. This category was added due to a number of expressed values that referenced what was needed to get things done, but without an explicit explanation of why those things needed to be done, or what their ultimate purpose would be. Because these values dealt with the need to get day-to-day tasks done, but did not reference broader goals (such as epistemic or ethical), we decided to place them in their own category.

## Footnotes

1 We use “research ethics” to refer specifically to ethics in research contexts, where the context might not distinguish between ethics in research and ethics or ethical principles more broadly. Otherwise, we use “ethics” in a general sense, which the context might further specify (as in “ethics in science”).

2 Values in this sense are not restricted to, but do include, specific “values” such as Schwartz’s (2004) principles that are the primary influences on decision-making, or cultural “values” as described in Sanderson et al. (2018).

3 For the purposes of this study, we treat theoretical virtues such as simplicity and understanding as epistemic values, broadly construed, while recognizing that for many other purposes these should be differentiated from truth and empirical adequacy.
